# Variation in multimorbidity estimates due to clinical coding: a cross-sectional study of 7.2 million adults in England

**DOI:** 10.1136/bmjph-2025-004120

**Published:** 2026-01-05

**Authors:** Tassella Isaac, Md Mehedi Hasan, Andrew Farmer, Hajira Dambha-Miller

**Affiliations:** 1 University of Southampton Faculty of Medicine, Southampton, UK; 2 University of Oxford, Oxford, England, UK

**Keywords:** Comorbidity, statistics and numerical data, Primary Prevention

## Abstract

**Objective:**

To examine how variation in clinical coding systems and the number of conditions included under different study criteria influence estimates of multimorbidity prevalence in a nationally representative adult population in England.

**Methods and analysis:**

We conducted a cross-sectional analysis of anonymised records from 7.2 million adults in the Clinical Practice Research Datalink, linked to Hospital Episode Statistics, covering the period from 1987 to 2020. Adults were included if they had at least two recorded health conditions. Multimorbidity was defined as ≥2 health conditions selected from a list of 54 conditions. Prevalence was estimated separately using general practice (GP) data, hospital data and combined sources, and stratified by age, sex, ethnicity and deprivation. A stepwise inclusion approach assessed the impact of expanding the number of conditions included after different study criteria. Gradient boosting (XGBoost) with Shapley Additive Explanations values identified predictors of multimorbidity recorded only in GP data. Directionality was examined using Pearson correlations.

**Results:**

Multimorbidity prevalence was 92.3% using GP data, 63.2% using hospital data and 100% when both sources were combined. Prevalence increased consistently as more conditions were included under different study criteria and was always higher in GP data. Discrepancies were most pronounced among younger adults and ethnic minority groups. GP-only coding was associated with younger age, female sex, shorter hospital stays, absence of Accident & Emergency use, no palliative care coding and lower deprivation.

**Conclusion:**

Estimates of multimorbidity prevalence are highly sensitive to both the clinical coding system used and the number of conditions included under different study criteria. Standardised approaches to condition selection and the integration of data sources are essential to ensure accurate measurement and equitable representation.

**WHAT IS ALREADY KNOWN ON THIS TOPIC**

- Multimorbidity is a growing global health challenge, yet its prevalence is inconsistently measured due to variation in the number of conditions included under different study criteria and the clinical coding systems used, such as International classification of diseases (ICD-10), systematised nomenclature of medicine clinical terms (SNOMED CT) and read coded clinical terms (READ).

**WHAT THIS STUDY ADDS**

- In a nationally representative cohort of 7.2 million adults in England with at least two recorded health conditions, we show that estimates of multimorbidity are highly dependent on the data source and the number of conditions included under different study criteria. General practice (GP) records consistently report higher prevalence than hospital data, with younger adults, women and ethnic minority groups disproportionately captured only in primary care.

**HOW THIS STUDY MIGHT AFFECT RESEARCH, PRACTICE OR POLICY**

- Variation in coding systems and number of conditions included under different study criteria can lead to substantial underestimation of multimorbidity and the exclusion of younger adults, women and ethnic minority groups. For clinicians, this highlights the need for consistent recording across care settings to ensure continuity and equity of care. For policymakers and health service planners, adopting standardised definitions and integrating primary and secondary care datasets are essential to accurately assess disease burden, allocate resources and design services that meet the needs of diverse populations.

## Introduction

 Multimorbidity, defined as the co-occurrence of two or more long-term conditions within an individual, is becoming increasingly common, driven by demographic ageing and improved survival from previously fatal diseases.^1,2^ Globally, around one-third of adults are estimated to live with multimorbidity, with prevalence exceeding 50% among those with chronic health conditions.^3^ In England, 54% of adults aged over 65 years were multimorbid in 2015, a figure projected to rise to 68% by 2035.^4^ Within the UK, multimorbidity drives more than half of all primary care consultations and accounts for approximately 70% of National Health Service (NHS) healthcare expenditure.^3,5^ The UK’s Major Conditions Strategy reports that people living with two or more long-term conditions account for more than half of NHS expenditure, including around 50% of hospital admissions, outpatient visits and primary care consultations. This equates to approximately £90 billion based on the 2022/23 NHS budget.^6^ Multimorbidity is associated with substantial clinical and system-level challenges, including greater complexity of care, increased healthcare use, higher treatment costs and poorer patient outcomes.^7^ In health systems that are often organised around single-disease models, the growing burden of multimorbidity necessitates more integrated and person-centred approaches to care delivery.

Accurately estimating multimorbidity prevalence at a population level is therefore essential for effective planning, resource allocation and monitoring of service needs. However, despite its centrality to public health and healthcare policy, there remains considerable uncertainty around how multimorbidity should be measured and reported. Current literature shows wide variation in the methods used to define and quantify multimorbidity. Studies differ in several key areas: the number and type of conditions included under different study criteria, the use of condition lists, the clinical coding systems applied and whether data are drawn from general practice (GP) care, hospital admissions or both.^8^ This heterogeneity directly affects prevalence estimates, undermining comparability across settings, populations and time periods. For example, the number of conditions included under different study criteria in published studies ranges from as few as two to as many as 285, with a median of 17.^9^ The selection of conditions is often guided by practical constraints, such as data availability, or by the adoption of condition lists developed in prior studies, many of which differ in scope, clinical relevance and intended use.^10^

Variation in clinical coding practices further complicates case identification. In England, for example, GP data are typically coded using READ or SNOMED CT systems, whereas hospital data are coded using ICD-10. Each system differs in structure, granularity and clinical context, potentially leading to misclassification or omission of certain conditions.^11^ Despite growing recognition of these issues, few studies have directly assessed how differences in clinical coding systems and the number of conditions included under different study criteria influence multimorbidity prevalence in large, linked datasets. Recent analysis of 172 563 participants in UK Biobank demonstrated poor agreement between GP care, hospital and baseline assessment data in identifying long-term conditions, with a median of only 4.7% of individuals captured across all three sources.^12^ A recent critical analysis further emphasised the absence of a universally accepted definition of multimorbidity and showed that prevalence estimates differ substantially depending on whether defined as ≥2 conditions, ≥3 conditions, across multiple body systems or as combined mental–physical conditions.^13^ Other studies highlight that the lack of a standardised definition continues to undermine comparability across studies and international surveillance, with approaches ranging from counts of conditions included under different study criteria to more complex biopsychosocial frameworks.^14^

In this study, we sought to address these gaps by examining how variation in clinical coding systems and the number of conditions included under different study criteria influence estimates of multimorbidity prevalence in a large, nationally representative population in England. Specifically, we used linked GP and hospital data to assess the impact of clinical coding system (READ, SNOMED CT and ICD-10) and the number of conditions included under different study criteria on reported multimorbidity prevalence. We also examined whether these differences varied by key demographic factors, including age, sex, ethnicity and socioeconomic status.

## Materials and methods

### Study design and data source

We conducted a cross-sectional analysis using anonymised electronic health records from the Clinical Practice Research Datalink (CPRD), a nationally representative dataset covering over 20 million individuals in the UK.^15,16^ Data were obtained from CPRD Gold, which uses READ codes mapped to SNOMED CT, and CPRD AURUM, which uses SNOMED CT directly^17^ (online supplemental table S1). Both were linked to Hospital Episode Statistics (HES), which records inpatient diagnoses using ICD-10. Duplicate records were removed. Clinical coding was harmonised across CPRD Gold and Aurum using CPRD’s code browser and the established READ-SNOMED CT mapping framework, enabling standard operational definitions of all conditions. Where READ did not map directly to a single SNOMED CT concept, we manually reviewed them and assigned the closest clinically appropriate equivalent (online supplemental table S3). Inclusion of both GP and hospital data enabled comparison of multimorbidity prevalence across healthcare settings and clinical coding systems. Socioeconomic status was measured using the Index of Multiple Deprivation (IMD), assigned at the area level based on residential postcode, and grouped into deciles. The IMD captures relative deprivation across multiple domains, including income, employment, education and health.^18^

### Study population

The study population comprised adults aged ≥18 years who were actively registered with a contributing GP during the study period (1 January 1987 to 31 December 2020). Individuals contributed follow-up from the date their practice met CPRD up-to-standard criteria. All included records had at least two recorded health conditions (multimorbidity). The CPRD population is broadly representative of the English population in terms of age, sex, ethnicity and deprivation.^15^

### Definition of multimorbidity

Multimorbidity was defined as the presence of two or more long-term conditions, based on a previously validated list of 59 conditions developed through national consensus.^19^ In this study, 54 conditions were used, as some conditions from the original list were unavailable in CPRD Gold and Aurum, and a small number were grouped due to low case numbers (online supplemental table S2). Each condition was flagged as present if a diagnostic code was recorded in either GP (READ/SNOMED CT) or hospital (ICD-10) data, with code lists standardised across systems to maintain consistency. Three datasets were constructed: (1) GP data: conditions identified using GP records (READ/SNOMED CT), (2) hospital data: conditions identified using hospital records (from HES) (ICD-10) and (3) combined data: conditions present in either GP or hospital data.

### Statistical analysis

The primary outcome was the prevalence of multimorbidity (≥2 conditions). To assess how prevalence varied with the number of conditions included under different study criteria, we performed a stepwise inclusion analysis for each data source (GP, hospital and combined), ranking conditions by prevalence and sequentially adding them until all 54 were included. At each step, prevalence with 95% CIs was estimated using the Wilson score method (without continuity correction), with results reported at selected thresholds of 2, 5, 10, 20 and 30 conditions for interpretability. Estimates were calculated separately for GP and hospital datasets, and differences between sources were tested using two-sample tests of proportions (p<0.05). Subgroup analyses were stratified by age, sex, ethnicity and IMD quintile; variation across sociodemographic groups was examined using χ^2^ tests, and prevalence compared between GP and hospital data using proportion tests.

To identify predictors of conditions recorded in GP but not hospital data, we trained a binary XGBoost classifier with the outcome gp_only=1 if a condition was documented in GP but absent from hospital data. Predictors included age, sex, ethnicity, IMD quintile, region, hospitalisation indicators, outpatient and A&E use, length of stay, palliative care status and HES match status. Categorical predictors were one-hot encoded. After excluding records with missing data, the model was trained on an 80/20 train–test split with parameters: objective = ‘binary: logistic’, eta=0.1, max_depth=4 and nrounds=100. Shapley Additive Explanations (SHAP) values quantified predictor contributions, and Pearson correlations between predictors and SHAP values were calculated to assess whether higher or lower values increased the likelihood of GP-only recording.

### Patient and public involvement

Patients and public were not involved in the design, conduct, reporting or dissemination plans of this study, as it used anonymised routinely collected electronic health records.

## Results

The study included 7 260 829 adults, of whom 55.2% were female, with a mean age of 54 years (SD=19) at time of multimorbidity diagnoses. Table 1 summarises the population characteristics.

**Table 1:** Baseline characteristics of adults aged ≥18 years with multimorbidity (≥2 long-term conditions) in the Clinical Practice Research Datalink (CPRD Gold and Aurum) linked to Hospital Episode Statistics (HES), 1987–2020 (N=7 260 829)

Characteristic	N (%) or mean (SD)
Total sample size	7 260 829
Age at index date, years	Mean (SD): 54 (19)
Age group	
18–39	1 571 427 (21.9%)
40–49	1 066 652 (14.9%)
50–59	1 366 128 (19%)
60–69	1 391 153 (19.4%)
70–79	1 105 747 (15.4%)
80+	670 405 (9.3%)
Sex	
Male	3 251 203 (44.8%)
Female	4 009 520 (55.2%)
Missing	106
Ethnicity	
White	5 995 453 (82.6%)
Mixed	43 894 (0.6%)
Asian	273 628 (3.8%)
Black	176 964 (2.4%)
Other	100 594 (1.4%)
Unknown	670 296 (9.2%)
Index of Multiple Deprivation (IMD)	
IMD 1 (least deprived)	1 392 155 (19.2%)
IMD 2	1 450 163 (20%)
IMD 3	1 422 513 (19.6%)
IMD 4	1 488 156 (20.5%)
IMD 5 (most deprived)	1 510 842 (20.8%)
Region	
Northeast	263 103 (3.6%)
Northwest	1 454 949 (20%)
Yorkshire and the Humber	295 671 (4.1%)
East Midlands	198 507 (2.7%)
West Midlands	1 132 204 (15.6%)
East of England	385 674 (5.3%)
Southwest	1 123 821 (15.5%)
South Central	1 442 990 (19.9%)
London	960 271 (13.2%)
Missing region	3639
Multimorbidity (≥2 conditions)	
From GP data (READ/SNOMED CT)	6 699 521 (92.3%)
From hospital data (ICD-10)	4 587 157 (63.2%)
From combined GP+hospital data	7 260 829 (100%)
Number of conditions	Median (IQR): 4 (4)
Ten most common conditions	
Hypertension	3 131 672 (43.1%)
Depression	2 830 412 (39%)
Osteoarthritis	2 673 532 (36.8%)
Anxiety	2 219 518 (32.8%)
Arrhythmia	2 235 723 (30.8%)
Drug and alcohol misuse	2 219 518 (30.6%)
Cancer	1 907 100 (26.3%)
Asthma	1 894 486 (26.1%)
Coronary heart disease	1 665 736 (23%)
Diabetes	1 377 031 (19%)
Healthcare utilisation	
HES matched records	7 204 100 (99.2%)
Ever admitted to hospital	6 113 433 (84.2%)
Attended A&E	4 983 542 (68.6%)
Attended outpatient appointment	6 270 520 (86.4%)
Received palliative care	785 663 (10.8%)

All data are derived from general practice data (READ/SNOMED CT; CPRD Gold and Aurum) and hospital data (ICD-10; HES). Multimorbidity was defined as the presence of two or more long-term conditions from a validated list of 54 conditions. Percentages are based on the total study population unless otherwise specified.

A&E, Accident & Emergency; GP, general practice.

Using the full set of 54 conditions, multimorbidity prevalence was 92.3% based on GP data (READ/SNOMED CT), 63.2% based on hospital data (ICD-10) and 100% when both data sources were combined.

### Impact of the number of conditions included under different study criteria

Multimorbidity prevalence increased sharply with the stepwise inclusion of more prevalent conditions and varied notably by data source. When only the top two conditions were included, prevalence was 10.2% for GP, 6.9% for hospital and 11.8% for combined data. Including the top five conditions increased prevalence to 51.6% (GP), 30.4% (hospital) and 61.1% (combined).

At 10 conditions, prevalence increased to 75% (GP), 46.5% (hospital) and 86% (combined). This trend continued with 20 conditions (GP: 86.6%; hospital: 58.5%; combined: 95%) and 30 conditions (GP: 89.9%; hospital: 61.6%; combined: 98.4%). Prevalence estimates were highly sensitive to both the clinical coding system and the number of conditions included under different study criteria, with combined data consistently yielding the highest prevalence. See figure 1 and table 2.

**Table 2:** Prevalence of multimorbidity (≥2 long-term conditions) among adults aged ≥18 years in CPRD Gold and Aurum linked to Hospital Episode Statistics (HES), 1987–2020 (n=7 260 829)

Number of conditions included	GP (%)	Hospital (%)	Combined (%)
2	10.2	6.9	11.8
5	51.6	30.4	61.1
10	75	46.5	86
20	86.6	58.5	95
30	89.9	61.6	98.4

Prevalence estimates are shown separately for general practice data (READ/SNOMED CT), hospital data (ICD-10) and combined general practice+hospital data. ‘Number of conditions included’ refers to how many of the most prevalent conditions from the study’s validated list were considered when calculating prevalence (here: the top 2, 5, 10, 20 and 30 of 54 conditions). In all analyses, multimorbidity is defined as the presence of ≥2 of the conditions included in the respective set.

GP, general practice.

**Figure 1:**
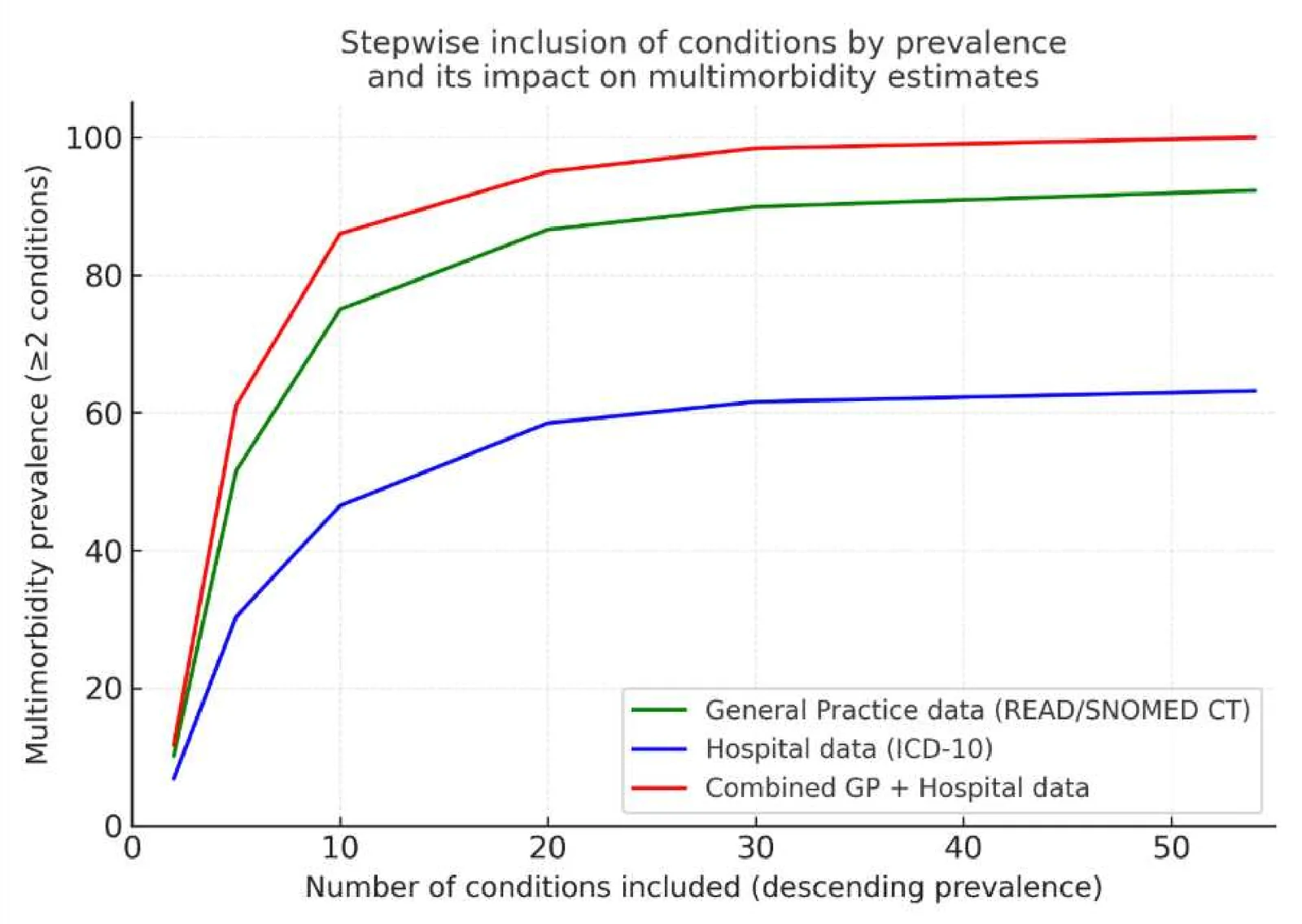
Stepwise inclusion of conditions by prevalence and its impact on multimorbidity estimates (≥2 long-term conditions). Prevalence of multimorbidity with increasing numbers of conditions included under different study criteria, ranked in descending order of prevalence. Lines represent prevalence based on general practice data (green, READ/SNOMED CT), hospital data (blue, ICD-10) and the combined dataset (red). The curves rise steeply with the inclusion of common conditions and plateau thereafter. Combined data consistently yield the highest estimates. Analysis includes adults aged≥18 years from CPRD Gold and Aurum linked to Hospital Episode Statistics (1987–2020). CPRD, Clinical Practice Research Datalink; GP, general practice.

### Prevalence by data source

The prevalence of multimorbidity also varied by clinical coding system (online supplemental table S4). Based on GP data (SNOMED/READ), 7 113 735 in the study population were classified as multimorbid, corresponding to a prevalence of 97.97% (95% CI 97.96% to 97.98%). Using hospital data (ICD-10), 5 736 329 were identified as multimorbid, giving a prevalence of 79% (95% CI 78.97% to 79.03%). The difference between GP and hospital estimates was statistically significant (p<0.001).

### Variation in multimorbidity prevalence by age, sex, IMD and ethnicity

Multimorbidity prevalence increased with age across all data sources. In GP records, prevalence ranged from 91.4% in the 18–39 age group to 93.8% in the 60–69 group. In hospital data, prevalence ranged from 43% in the 18–39 group to 72.7% in the 60–69 group. All differences between GP and hospital estimates were statistically significant (p<0.001).

Among males, 91.5% were multimorbid based on GP data (95% CI 91.49% to 91.55%) compared with 64.5% in hospital data (95% CI 64.40% to 64.51%). Among females, GP-based prevalence was 92.5% (95% CI 92.48% to 92.53%) and hospital-based prevalence was 62.1% (95% CI 62.09% to 62.19%). Differences between GP and hospital were statistically significant (p<0.001).

By deprivation, GP-based prevalence decreased from 92.5% in the least deprived quintile (IMD 1) to 91.5% in the most deprived quintile (IMD 5). Hospital-based prevalence increased with deprivation, from 61.2% in IMD 1 to 65.6% in IMD 5. Differences between GP and hospital estimates were statistically significant in each quintile (p<0.001).

Across ethnic groups, GP-based prevalence ranged from 89.6% (‘Other’) to 92.5% (Asian). Hospital-based prevalence ranged from 29.4% (unknown) to 67.8% (White). The largest differences between GP and hospital estimates were observed in the ‘Other’ group (89.6% vs 51.4%) and in Black ethnicity (91.3% vs 54.0%). All GP versus hospital comparisons were statistically significant (p<0.001) (online supplemental figure S1, table 3).

**Table 3:** Prevalence of multimorbidity (≥2 long-term conditions) by demographic subgroup and data source

Subgroup	Group	N	Prevalence (GP)	95% CI (GP)	Prevalence (hospital)	95% CI (hospital)	Prevalence (combined)	95% CI (combined)	P (GP vs hospital)
Age group	18–39	1 571 427	91.4%	91.3% to 91.4%	43%	42.9% to 43.1%	100%	>99.9% to 100%	<0.001
	40–49	1 066 652	92.8%	92.8% to 92.9%	54%	53.9% to 54.1%	100%	>99.9% to 100%	<0.001
	50–59	1 366 128	93.5%	93.4% to 93.5%	62.8%	62.7% to 62.9%	100%	>99.9% to 100%	<0.001
	60–69	1 391 153	93.8%	93.8% to 93.9%	72.7%	72.6% to 72.8%	100%	>99.9% to 100%	<0.001
	70–79	1 105 747	92.6%	92.6% to 92.7%	79.5%	79.4% to 79.6%	100%	>99.9% to 100%	<0.001
	80+	670 405	84.1%	84.0% to 84.2%	79.9%	79.8% to 80%	100%	>99.9% to 100%	<0.001
Sex	Male	3 251 203	91.5%	91.5%–91.6%	64.5%	64.4% to 64.5%	100%	>99.9% to 100%	<0.001
	Female	4 009 520	92.5%	92.4%–92.5%	62.1%	62.1% to 62.2%	100%	>99.9% to 100%	<0.001
IMD quintile	1 (least)	1 392 155	92.5%	92.4%–92.5%	61.2%	61.1% to 61.3%	100%	>99.9% to 100%	<0.001
	2	1,450, 163	92.3%	92.3%–92.4%	62.7%	62.7% to 62.8%	100%	>99.9% to 100%	<0.001
	3	1 422 513	92.2%	92.1%–92.2%	63%	63% to 63.1%	100%	>99.9% to 100%	<0.001
	4	1 488 156	91.8%	91.8%–91.9%	63.1%	63.1% to 63.2%	100%	>99.9% to 100%	<0.001
	5 (most)	1 510 842	91.5%	91.4%–91.5%	65.6%	65.5% to 65.7%	100%	>99.9% to 100%	<0.001
Ethnicity	White	5 995 453	92.1%	92%–92.1%	67.8%	67.7% to 67.8%	100%	>99.9% to 100%	<0.001
	Asian	273 628	92.5%	92.4%–92.6%	56.7%	56.5% to 56.9%	100%	>99.9% to 100%	<0.001
	Black	176 964	91.3%	91.2%–91.4%	54%	53.7% to 54.2%	100%	>99.9% to 100%	<0.001
	Mixed	43 894	90.6%	90.3%–90.8%	51.5%	51.1% to 52%	100%	>99.9% to 100%	<0.001
	Other	100 594	89.6%	89.4%–89.8%	51.4%	51.1% to 51.7%	100%	>99.9% to 100%	<0.001
	Unknown	670 296	92.4%	92.3%–92.4%	29.4%	29.3% to 29.5%	100%	>99.9% to 100%	<0.001

The table reports prevalence estimates of multimorbidity among adults aged ≥18 years in the Clinical Practice Research Datalink (CPRD Gold and Aurum) linked to Hospital Episode Statistics (HES) (1987–2020). Estimates are shown separately for general practice data (READ/SNOMED CT; CPRD Gold and Aurum), hospital data (ICD-10; HES), and combined general practice+hospital data, with 95% CIs. Subgroups include age, sex, Index of Multiple Deprivation (IMD) quintile and ethnicity. Differences between general practice and hospital prevalence were tested using two-sample proportion tests (p values shown).

GP, general practice.

### Predictors of gap in clinical coding

Among the variables examined, duration of hospitalisation after being diagnosed with multimorbidity was the strongest predictor of whether a condition was only documented in GP data and not in hospital, followed by age at index, A&E attendance and sex (female). Online supplemental figure S2 visualises the top predictors based on mean absolute SHAP values. Variables contributing less than 0.04 were excluded for interpretability. To examine directionality, we calculated Pearson correlation coefficients between each predictor and its corresponding SHAP values. This allowed us to determine whether higher or lower values of each feature increased the likelihood of GP-only documentation. Conditions were more often captured in GP data but missed in hospital data among younger individuals (r = –0.77), females, those with shorter hospital stays (r = –0.40), no A&E attendance (r = –0.84), not receiving palliative care (r = –0.94) and from less deprived areas (r = –0.94). All associations were statistically significant (p<0.01) (figure 2).

**Figure 2:**
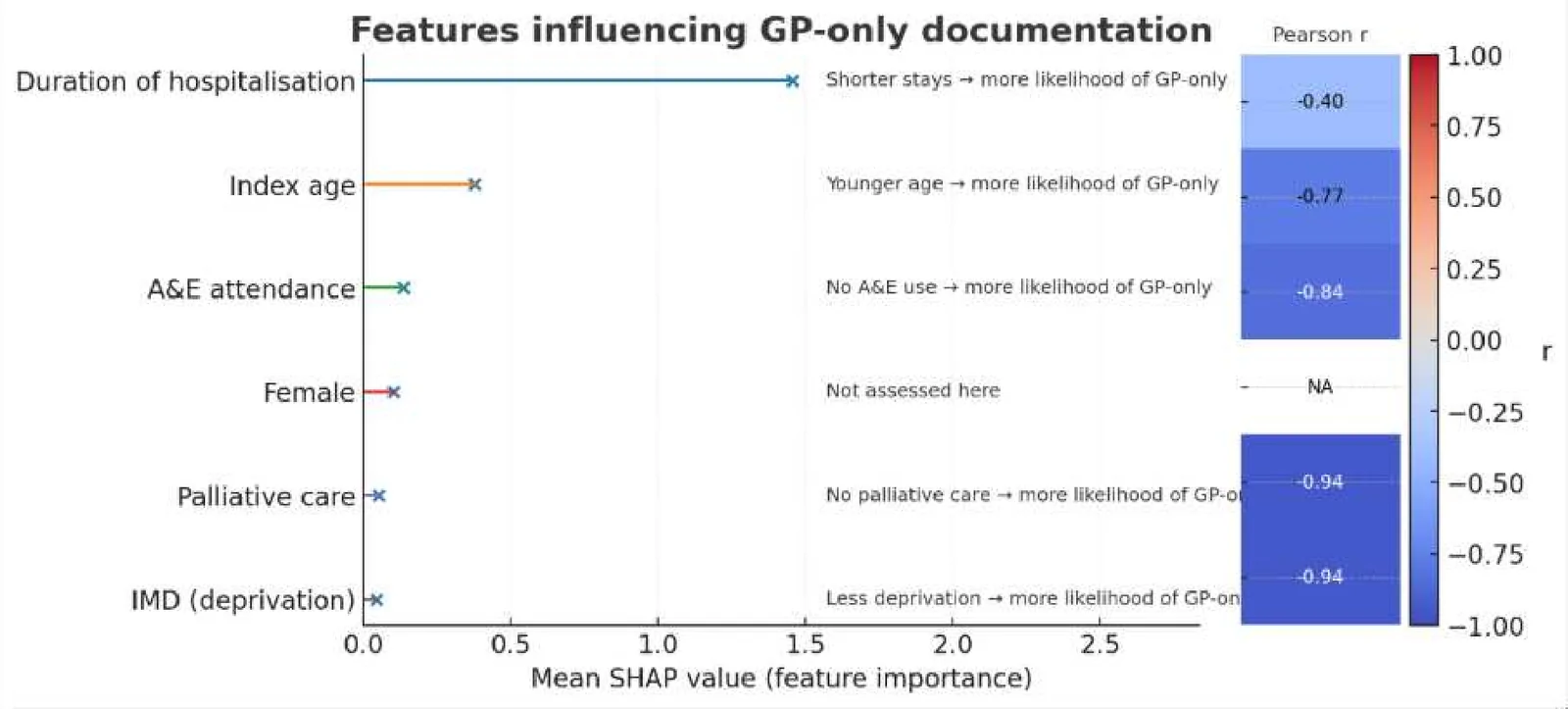
Predictors of ‘general practice (GP)-only’ documentation of multimorbidity (≥2 long-term conditions) among adults aged ≥18 years in the Clinical Practice Research Datalink (CPRD Gold and Aurum) linked to Hospital Episode Statistics (HES) (1987–2020). Predictors are ranked by mean absolute SHAP value from an XGBoost model of GP-only recording, defined as a condition documented in GP data (READ/SNOMED CT; CPRD Gold and Aurum) but not in hospital data (ICD-10; HES). Direction of association is indicated by Pearson correlation coefficients (**r**) between each predictor and its SHAP values; negative r indicates that lower values of the predictor are associated with greater likelihood of GP-only recording. Sex (female) is categorical and therefore shown without a correlation value. CPRD, Clinical Practice Research Datalink; IMD, Index of Multiple Deprivation; SHAP, Shapley Additive Explanations.

## Discussion

In this study, we aimed to examine how variation in clinical coding systems and the number of conditions included under different study criteria influence estimates of multimorbidity prevalence in a nationally representative adult population in England. Using linked GP data (READ/SNOMED CT) and hospital data (ICD-10) for over 7.2 million adults in CPRD, we found that prevalence estimates are highly sensitive to both the clinical coding system and the number of conditions included under different study criteria.

Multimorbidity prevalence was consistently higher in GP data (92.3%) than in hospital data (63.2%). This likely reflects the broader scope of SNOMED CT, which enables longitudinal documentation of health conditions, compared with ICD-10, which is structured around episodic, billing-focused encounters.^20–26^ Given that these figures represent adults with at least two recorded conditions, the absolute prevalence is expectedly high; however, the large discrepancy with hospital data highlights how hospital data alone may underestimate disease burden. These findings are consistent with previous research showing that reliance on a single data source can underestimate disease burden. For example, the sensitivity of cancer diagnoses in CPRD and hospital data has been shown to vary substantially by site and source.^27^

The number of conditions included under different study criteria also had a major impact on prevalence estimates. When only the two most common conditions were included, prevalence ranged from 6.9% (hospital) to 11.8% (combined GP+hospital). Including the top five conditions increased prevalence to 61.1%, and when the top 30 conditions were included, prevalence reached 98.4%.

Sociodemographic variation was also observed. Consistent with prior research,^28^ multimorbidity prevalence increased with age in both datasets; both estimates were consistently higher in GP data. For example, prevalence among those aged 18–39 was 91.4% in GP data compared with 43% in hospital data, rising to 93.8% and 72.7% respectively among those aged 60–69. These differences likely reflect care patterns, with younger people and those with less severe conditions more often managed in GP alone.

Sex-based differences were modest. Women had a slightly higher prevalence in GP data (92.5% vs 91.5% in men) and slightly lower in hospital data (62.1% vs 64.5%). This aligns with evidence that women have greater GP utilisation, partly due to reproductive health needs.^29–33^ Socioeconomic gradients differed by source: in GP data, prevalence declined slightly with increasing deprivation (from 92.5% to 91.5%), whereas in hospital data it increased (from 61.2% to 65.6%). This may indicate disparities in access to GP services or under-recording in more deprived areas, alongside higher hospitalisation rates due to more advanced or severe disease.^34^

We also found ethnic disparities. Prevalence was highest in people of White ethnicity across both sources, but the gap between sources was wider among minority ethnic groups, for example, 37.3% in people of Black ethnicity and 38.2% in those recorded as ‘Other’. These differences may reflect variation in care-seeking behaviour, access or recording practices and highlight the need for improved interoperability between coding systems.^35^

Finally, we examined predictors of discordant documentation between sources. SHAP analysis of an XGBoost model identified younger age, female sex, fewer hospital admissions, shorter stays, absence of A&E use or palliative care and residence in less deprived areas as factors associated with conditions recorded in GP data but missing in hospital data. This suggests that lower hospital utilisation is a major driver of under-recording in hospital data and therefore represents a missed opportunity to develop policies and services that support prevention and proactive early care in these population groups.

### Strengths and limitations

We used a large, nationally representative cohort of over 7.1 million adults in England, providing reliable statistical power and generalisability. The use of a linked GP and hospital dataset allowed direct comparison of clinical coding systems across healthcare settings, improving completeness of condition ascertainment. The structured stepwise inclusion of conditions enabled assessment of how prevalence varies with the number of conditions included under different study criteria, addressing a recognised gap in the literature. Stratification by age, sex, ethnicity and socioeconomic status revealed important disparities across subgroups, including those often underrepresented in research. The application of machine learning (XGBoost) with SHAP values provided further insight into predictors of discrepancies in condition documentation.

Limitations included differences in clinical coding practices between systems, which may lead to misclassification. ICD-10 codes in hospital data may under-represent conditions predominantly managed in GP data, which could bias comparisons. In addition, READ codes and SNOMED CT do not map 1:1 and differ in structure and granularity. SNOMED CT is a polyhierarchical ontology, whereas READ is a linear classification system. As a result, direct mapping may lead to incomplete alignment of diagnostic categories and introduce misclassification.^36^ The non-inclusion of Office of Population Censuses and Surveys Classification of Interventions and Procedures (OPSC-4) procedure codes in defining health conditions represents a further limitation. Although primarily procedural, these codes can support identification of conditions in secondary care, and their omission may have contributed to under-representation.^37^ Moreover, the mapping of READ codes to our definition of multimorbidity was based on the number of conditions included under different study criteria, without accounting for severity, which may limit its applicability for care prioritisation. Estimates also remain sensitive to the number of conditions included. Finally, the exclusion of records with missing data from the machine learning analysis may introduce bias if excluded individuals differ systematically from those included.

## Conclusion and future implications

This study shows that multimorbidity prevalence estimates vary depending on the coding system, data source and the number of conditions included under different study criteria. Use of a single clinical coding system, particularly one designed for episodic or administrative purposes, such as ICD-10, may underestimate multimorbidity and provide an incomplete picture of population health. Given that the median number of conditions per person was four, these results show that both clinical and policy decisions are contingent on the definitional scope. This supports calls for standardised thresholds, such as ≥12 conditions, to enable more consistent identification of people with complex needs.^33,38–40^

In practice, hospital data alone may not capture the full extent of multimorbidity, particularly for people managed predominantly in GP. This has implications for risk stratification, care planning and resource allocation, which often prioritise secondary care. Integrated use of GP and hospital data could enable more accurate identification of people with complex needs, support earlier intervention and improve continuity of care across settings. Future research should also explore inconsistencies in coding, especially in hospital data, to improve data quality and completeness. Work is also needed to examine underrepresented groups, such as younger adults, minority ethnic populations and those with infrequent hospital visits, who may be missed in hospital-based estimates but still require coordinated care. Wider adoption of linked datasets that combine SNOMED CT/READ and ICD-10 coding systems would enable more accurate estimation of multimorbidity and better inform clinical guidelines, service design and equitable policy development.

Looking ahead, improvements in multimorbidity surveillance will require addressing long-standing challenges at the primary-secondary care interface. As highlighted in the national guidance, greater interoperability between GP and hospital systems, including shared access to clinical records and improved digital pathways such as Advice & Guidance, could reduce fragmentation in diagnostic information and support more complete identification of health conditions.^41,42^ Strengthening multidisciplinary collaboration and joint coding governance across sectors would help reduce variation in recording practices, particularly for conditions represented unevenly across READ, SNOMED CT and ICD-10.^43^ While statistical methods cannot fully correct for structural imbalance in the volume and granularity of diagnostic codes, future research could examine ontology-based clustering, probabilistic mapping approaches or weighted-case definitions to reduce the effects of disproportionate code sets on multimorbidity estimates.

## Supplementary material

10.1136/bmjph-2025-004120 online supplemental file 1

## Data Availability

Data may be obtained from a third party and are not publicly available.
