# Supplementary material for: Variation in multimorbidity estimates due to clinical coding: a cross-sectional study of 7.2 million adults in England
Source: BMJ Public Health. 2026 Jan 5;4(1):e004120. doi: 10.1136/bmjph-2025-004120 (PMC13663701; doi:10.1136/bmjph-2025-004120)
Supplement: Supplementary file 1 [file bmjph-4-1-s001.docx]

**Supplementary Material**

**Table S1.** READ and SNOMED CT codes used to identify 54 long-term conditions (LTCs) among adults aged ≥18 years in CPRD Gold and Aurum, 1987–2020 (N = 7,260,829).

Codes were extracted from GP data within the Clinical Practice Research Datalink (CPRD) to define long-term conditions contributing to multimorbidity.

[54 LTC Codes](https://view.officeapps.live.com/op/view.aspx?src=https%3A%2F%2Fraw.githubusercontent.com%2Fbig-data-in-health%2FCPRD%2Frefs%2Fheads%2Fmain%2FMedical_Codes%2F59LTC_CPRD.xlsx&wdOrigin=BROWSELINK)

**Table S2.** Long-term conditions (LTCs) identified among adults aged ≥18 years in CPRD Gold and Aurum, 1987–2020 (N = 7, 260, 829), mapped against the original 59 LTCs defined through national consensus.

Conditions were grouped where required to reflect availability in CPRD data, resulting in 54 LTCs used to define multimorbidity in this study.

|  | **Long-Term Conditions in the Original 59**  **Conditions** | **Long-Term Conditions Present in The Study** |
| --- | --- | --- |
| 1 | Addison’s Disease | Yes |
| 2 | Anaemia | Yes  Present as Pernicious anaemia |
| 3 | Anxiety | Yes |
| 4 | Depression | Yes |
| 5 | Congenital Heart Disease | Yes |
| 6 | Chromosomal Abnormality | Yes |
| 7 | Bipolar Disorder | Yes |
| 8 | Schizophrenia | Yes |
| 9 | Chronic Liver Disease | Yes |
| 10 | Alcohol-related Liver Disease | Merged and renamed as: Chronic  Liver Disease and Alcoholic  Liver Disease |
| 11 | Aortic Aneurysm | Yes |
| 12 | Arrhythmia | Yes |
| 13 | Asthma | Yes |
| 14 | Autism | Yes |
| 15 | Bronchiectasis | Yes |
| 16 | Cancer | Yes |

| 17 | Cerebral Benign Tumours | No |
| --- | --- | --- |
| 18 | Chronic Back Pain | No |
| 19 | Chronic Lyme Disease | Yes |
| 20 | Chronic Pancreatitis | Yes |
| 21 | Chronic Kidney Disease stage 3-5 | Yes |
| 22 | Chronic Pain | Yes |
| 23 | Chronic Obstructive Pulmonary Disease | Yes |
| 24 | Chronic Urinary Tract Infections | Yes |
| 25 | Connective Tissue Disease | Yes |
| 26 | Coronary Heart Disease | Yes |
| 27 | Heart Failure | Yes |
| 28 | Hypertension | Yes |
| 29 | Cystic Fibrosis | Yes |
| 30 | Dementia | Yes |
| 31 | Diabetes | Yes |
| 32 | Drug or Alcohol Misuse | Yes |
| 33 | Eating Disorders | Yes |
| 34 | Endometriosis Adenomyosis | Yes |
| 35 | Epilepsy | Yes |
| 36 | Gout | Yes |
| 37 | Hearing Loss | Yes |
| 38 | Hemi/Para/Quadriplegia | No |
| 39 | HIV/AIDS | Yes |
| 40 | Inflammatory Bowel Disease | Yes |
| 41 | Long COVID | Yes  Present as Post-Acute COVID  19 |
| 42 | Meniere’s Disease | Yes |
| 43 | Multiple Sclerosis | Yes |
| 44 | Musculoskeletal Injury | Yes |
| 45 | Osteoarthritis | Yes |
| 46 | Osteoporosis | Yes |
| 47 | Parkinson’s Disease | Yes |
| 48 | Peptic Ulcer Disease | Yes |
| 49 | Peripheral Neuropathy | Yes |
| 50 | Peripheral Vascular Disease | Yes |
| 51 | Post-Traumatic Stress Disorder | Yes |
| 52 | Sickle Cell Disease | No |
| 53 | Stroke | Yes |
| 54 | Thyroid Disease | Yes |
| 55 | Transient Ischemic Attack | Yes |
| 56 | Tuberculosis | Yes |
| 57 | Valvular Diseases | Yes  Present as Heart Valvular  Disorder |
| 58 | Venous Thromboembolism | Yes |
| 59 | Visual Impairment | Yes |

**Table S3.** Mapping Coverage between READ and SNOMED-CT Concepts for Health Conditions in CPRD.

Mapping completeness was calculated as:
(Number of READ codes with at least one SNOMED CT match ÷ Total READ codes) × 100%.

|  | **Condition** | **READ codes used** | **SNOMED codes used** | **Successfully mapped (%)** | **Unmapped (%)** |
| --- | --- | --- | --- | --- | --- |
| 1 | Addison’s Disease | 16 | 14 | 93.8% | 6.3% |
| 2 | Anaemia | 5 | 4 | 100% | 0% |
| 3 | Anxiety | 70 | 57 | 93.2% | 6.8% |
| 4 | Depression | 139 | 92 | 100% | 0% |
| 5 | Congenital Heart Disease | 281 | 181 | 100% | 0% |
| 6 | Chromosomal Abnormality | 23 | 13 | 100% | 0% |
| 7 | Bipolar Disorder | 132 | 86 | 100% | 0% |
| 8 | Schizophrenia | 92 | 51 | 99.1% | 0.9% |
| 9 | Chronic Liver Disease | 104 | 77 | 100% | 0% |
| 10 | Aortic Aneurysm | 77 | 45 | 59.8% | 40.2% |
| 11 | Arrhythmia | 331 | 243 | 98.2% | 1.8% |
| 12 | Asthma | 132 | 155 | 100% | 0% |
| 13 | Autism | 13 | 13 | 100% | 0% |
| 14 | Bronchiectasis | 7 | 6 | 100% | 0% |
| 15 | Cancer | 2484 | 2008 | 100% | 0% |
| 16 | Chronic Lyme Disease | 8 | 8 | 75% | 25% |
| 17 | Chronic Pancreatitis | 7 | 7 | 100% | 0% |
| 18 | Chronic Kidney Disease | 41 | 41 | 77.3% | 22.7% |
| 19 | Chronic Pain | 13 | 10 | 100% | 0% |
| 20 | COPD | 100 | 74 | 69.4% | 30.6% |
| 21 | Connective Tissue Disease | 124 | 97 | 100% | 0% |
| 22 | Coronary Heart Disease | 210 | 149 | 95.8% | 4.2% |
| 23 | Heart Failure | 117 | 117 | 98.3% | 1.7% |
| 24 | Hypertension | 108 | 108 | 98.7% | 1.3% |
| 25 | Cystic Fibrosis | 18 | 18 | 100% | 0% |
| 26 | Dementia | 102 | 102 | 100% | 0% |
| 27 | Diabetes | 229 | 229 | 99.1% | 0.9% |
| 28 | Drug or Alcohol Misuse | 407 | 407 | 99.7% | 0.3% |
| 29 | Eating disorders | 31 | 29 | 100% | 0% |
| 30 | Endometriosis | 36 | 36 | 100% | 0% |
| 31 | Epilepsy | 105 | 105 | 98.2% | 1.8% |
| 32 | Gout | 45 | 45 | 98.4% | 1.6% |
| 33 | Hearing Loss | 80 | 58 | 83.3% | 16.7% |
| 34 | HIV/AIDS | 55 | 55 | 97.4% | 2.6% |
| 35 | Inflammatory Bowel Disease | 27 | 27 | 100% | 0% |
| 36 | Long COVID | 4 | 0 | 0% | 0% |
| 37 | Ménière’s Disease | 9 | 9 | 100% | 0% |
| 38 | Multiple Sclerosis | 24 | 24 | 96% | 4% |
| 39 | Musculoskeletal Injury | 458 | 564 | 95% | 5% |
| 40 | Osteoarthritis | 198 | 194 | 100% | 0% |
| 41 | Osteoporosis | 55 | 55 | 100% | 0% |
| 42 | Parkinson’s Disease | 5 | 5 | 100% | 0% |
| 43 | Peptic Ulcer Disease | 139 | 139 | 100% | 0% |
| 44 | Peripheral Neuropathy | 90 | 90 | 100% | 0% |
| 45 | Peripheral Vascular Disease | 245 | 245 | 99.6% | 0.4% |
| 46 | PTSD | 2 | 2 | 100% | 0% |
| 47 | Stroke | 110 | 110 | 99.4% | 0.6% |
| 48 | Thyroid disease | 84 | 84 | 100% | 0% |
| 49 | Transient Ischaemic attack | 14 | 14 | 100% | 0% |
| 50 | Tuberculosis | 200 | 200 | 100% | 0% |
| 51 | Valvular Disease | 365 | 365 | 100% | 0% |
| 52 | Venous Thromboembolism | 58 | 58 | 100% | 0% |
| 53 | Visual Impairment | 58 | 58 | 100% | 0% |
| 54 | Metastatic Cancer | 11 | 11 | 100% | 0% |

**Table S4.** Prevalence of multimorbidity (≥2 long-term conditions) among adults aged ≥18 years in CPRD Gold and Aurum linked to Hospital Episode Statistics, 1987–2020 (N = 7,260,829).

Estimates are shown separately for GP data (READ/SNOMED CT), hospital data (ICD-10), and combined GP + hospital data. Ninety-five percent confidence intervals were calculated using the Wilson score method.

| **Source** | **Cases** | **Prevalence (%)** | **95% CI** |
| --- | --- | --- | --- |
| GP (SNOMED/READ) | 7,113, 735 | 97.97 | 97.96-97.98 |
| HES (ICD-10) | 5, 736, 329 | 79 | 78.97-79.03 |
| Combined | 7, 260, 829 | 100 | 99.99-100 |

**Figure S1.** Variation in prevalence of multimorbidity (≥2 long-term conditions) among adults aged ≥18 years in CPRD Gold and Aurum linked to Hospital Episode Statistics, 1987–2020 (N = 7,260,829).

Prevalence estimates are shown separately for GP data (READ/SNOMED CT), hospital data (ICD-10), and combined GP + hospital data, stratified by demographic subgroups: (A) age group, (B) sex, (C) Index of Multiple Deprivation (IMD) quintile, and (D) ethnicity.

**Figure S2.** SHAP feature importance for predictors of GP-only documentation of multimorbidity (≥2 long-term conditions) among adults aged ≥18 years in CPRD Gold and Aurum linked to Hospital Episode Statistics, 1987–2020 (N = 7,260,829).

Bars show the mean absolute SHAP value for each predictor in the XGBoost model, indicating its average contribution to the model output. Duration of hospitalisation and age at index were the most influential predictors. Predictors with mean |SHAP| values <0.04 were excluded for clarity.
